# First report of *in vitro* selection of RNA aptamers targeted to recombinant *Loxosceles laeta* spider toxins

**DOI:** 10.1186/0717-6287-47-2

**Published:** 2014-03-26

**Authors:** Amalia Sapag, Catalina Salinas-Luypaert, Carlos Constenla-Muñoz

**Affiliations:** Laboratory of Gene Pharmacotherapy, Department of Pharmacological and Toxicological Chemistry, Faculty of Chemical and Pharmaceutical Sciences, Universidad de Chile, Sergio Livingstone Pohlhammer 1007, Independencia, Santiago, 838-0492 Chile

**Keywords:** Antivenom, *Loxosceles laeta*, RNA aptamers, SELEX, SMD-Ll1, SMD-Ll2, Sphingomyelinase D

## Abstract

**Background:**

Loxoscelism is the envenomation caused by the bite of *Loxosceles spp.* spiders. It entails severe necrotizing skin lesions, sometimes accompanied by systemic reactions and even death. There are no diagnostic means and treatment is mostly palliative. The main toxin, found in several isoforms in the venom, is sphingomyelinase D (SMD), a phospholipase that has been used to generate antibodies intended for medical applications. Nucleic acid aptamers are a promising alternative to antibodies. Aptamers may be isolated from a combinatorial mixture of oligonucleotides by iterative selection of those that bind to the target. In this work, two *Loxosceles laeta* SMD isoforms, Ll1 and Ll2, were produced in bacteria and used as targets with the aim of identifying RNA aptamers that inhibit sphingomyelinase activity.

**Results:**

Six RNA aptamers capable of eliciting partial but statistically significant inhibitions of the sphingomyelinase activity of recombinant SMD-Ll1 and SMD-Ll2 were obtained: four aptamers exert ~17% inhibition of SMD-Ll1, while two aptamers result in ~25% inhibition of SMD-Ll2 and ~18% cross inhibition of SMD-Ll1.

**Conclusions:**

This work is the first attempt to obtain aptamers with therapeutic and diagnostic potential for loxoscelism and provides an initial platform to undertake the development of novel anti *Loxosceles* venom agents.

## Background

The envenomation resulting from the bite of spiders of the genus *Loxosceles* is called loxoscelism [1–3]. It is characterized by severe necrotizing wounds in the skin (cutaneous loxoscelism), which are sometimes accompanied by systemic reactions that can lead to death (viscerocutaneous loxoscelism). A distinctive skin ulcer develops rapidly, within days, but may take months to heal leaving behind disfiguring scars. In the more severe systemic form of poisoning, which has a higher incidence in children, intravascular hemolysis may cause anemia and acute renal failure within 72 hours. There are no diagnostic means available and treatment is mainly palliative.

*Loxosceles* spiders are mostly found in the warm and tropical climate areas of the Americas. *Loxosceles reclusa* (brown recluse spider) and *L. deserta* account for most cases of loxoscelism in the United States. In South America, the three major species responsible for envenomation are *L. intermedia*, *L. gaucho* and *L. laeta*[2]. The frequency of viscerocutaneous loxoscelism is higher (~15%) in geographic areas where *L. laeta* is the predominant species, such as Chile [4], Peru, and certain regions of Brazil [5]. The actual incidence of envenomation is not known because the spider is seldom captured and presented at the time of medical evaluation.

The venom of *Loxosceles* spiders contains several toxins [6], including multiple isoforms of sphingomyelinase D (SMD) [7], a phospholipase capable of eliciting biological damage similar to that of whole venom, *i.e.*, dermonecrosis, platelet aggregation and hemolysis [7–9]. Sphingomyelinase D has two known substrates: sphingomyelins, which are abundant in cell membranes —especially in skin and erythrocytes— and give rise to phosphoceramides, and lysophosphatidylcholine, a substrate abundant in plasma, which generates lysophosphatidic acid, a pleiotropic mediator [10]. A number of *Loxosceles* SMDs have been cloned, sequenced and characterized, such as several from *L. laeta*[8, 11–13], *L. intermedia*[14], *L. reclusa* and *L. boneti*[15].

Although there are several *Loxosceles* antivenoms commercially available [2, 16], their safety and efficacy remain controversial. Given that equine hyperimmune sera may cause allergic reactions, therapeutic antibodies obtained against recombinant SMDs [13, 17] are under preclinical development. For example, an antiserum raised against a toxic recombinant SMD from *L. laeta* can block the skin lesion caused by whole *L. laeta* venom in rabbits [8]; similar immunoprotection using a nontoxic recombinant SMD has been reported [11]. Monoclonal antibodies are also capable of neutralizing dermonecrotic and lethality effects of *Loxosceles* venoms but do not always confer cross species protection [18, 19]. Antigenic cross reactivity [*e.g.,*[13, 17, 20–22] is relevant to the development of wide spectrum *Loxosceles* antivenoms. Although significant reactivity and dermonecrotic protection has been reported between some species, Olvera *et al.*[13] suggest that the sequence divergence of the *L. laeta* SMDs (*e.g.*, only 40% identity with *L. boneti* SMD) may account for their lack of *in vitro* and *in vivo* responsiveness to cross species antisera. Thus, treatment for envenomation by *L. laeta* requires special attention.

**Figure 1:**
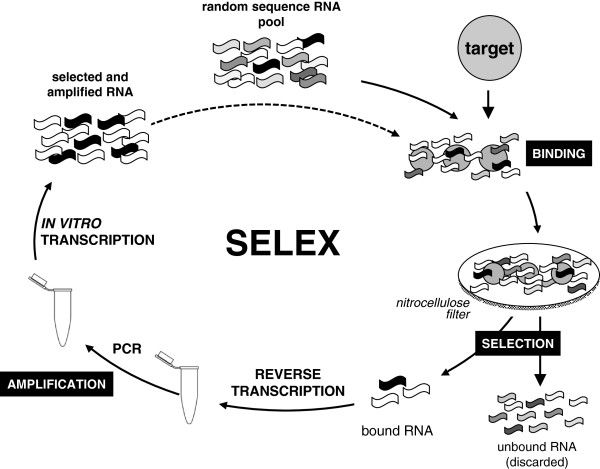
**Iterative** ***in vitro*** **selection of RNA aptamers (SELEX).**

In spite of their widespread use in therapeutics, antibodies have limitations such as the requirement of live animals for their development, immunogenicity, limited stability, and elevated costs. Nucleic acid aptamers overcome these limitations and are thus a promising alternative to antibodies. RNA or DNA oligonucleotides that have been selected for their ability to bind to other molecules with high affinity and high specificity have great applicability in therapeutics [23]. In particular, an aptamer having the capacity to bind to a *Loxosceles* sphingomyelinase D and abolish its enzymatic activity would have potential as an antidote for loxoscelism. Aptamers may be obtained by an iterative *in vitro* selection strategy (Figure 1) known as “systematic evolution of ligands by exponential enrichment” (SELEX) (reviewed by Stoltenburg *et al.*[24]). An oligonucleotide library of ≥10^13^ different sequences, typically ≤100 nucleotides long, is subjected to *in vitro* selection based on the desired biological activity, usually the affinity for a particular target. The molecules which satisfy the selection criterion are separated from those that do not and, following an amplification step, are subjected to a new selection round; this cycle is usually performed 4–20 times. Amplification is done by the polymerase chain reaction (PCR) or, in the case of RNA aptamers, by reverse transcription, PCR, and *in vitro* transcription. Finally, aptamers are cloned, sequenced, and tested individually for binding or other biological activity. In this work, RNA aptamers selected for binding to *L. laeta* SMD isoforms Ll1 and Ll2 [13], which differ in 47 of 285 amino acids, were obtained and analyzed for their capacity to inhibit sphingomyelinase activity.

## Results

### Selection of aptamers capable of binding to *Loxosceles laeta* SMDs

RNAs that bind to either isoform Ll1 or Ll2 of sphingomyelinase D were obtained by selection from starting random RNA pools having ~10^13^ different sequences. For SMD-Ll1, two independent iterative selection (SELEX) processes, E and F, were performed at protein:RNA ratios of 1:10 and 1:5 using 10 or 20 pmoles of SMD-Ll1 respectively and 100 pmoles of RNA. Identical initial pools of RNA (named s0: selected 0 times) were used for E and F. Twelve selection cycles were carried out for SELEX E and ten for SELEX F. For SMD-Ll2, an iterative selection of 12 cycles, called SELEX G, was performed at a protein:RNA ratio of 1:10 using 10 pmoles of SMD-Ll2. The RNA molecules of the initial pool (s0) were different from those used for E and F.

### Inhibition of sphingomyelinase activity by pools of selected RNA

**Figure 2:**
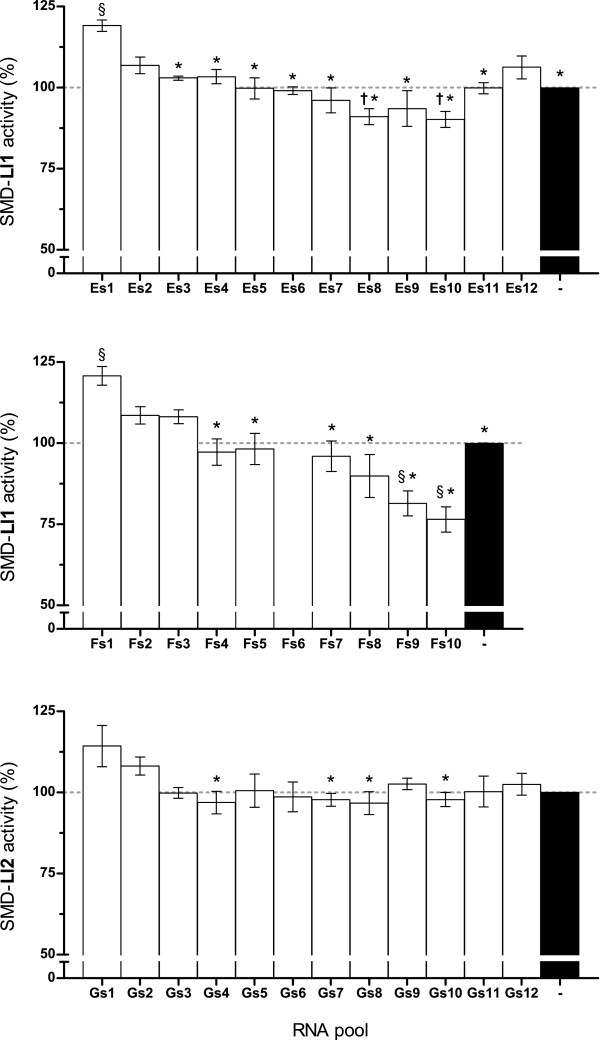
**Effect of pools of RNA selected for binding to SMD-Ll1 (SELEX E and SELEX F) or to SMD-Ll2 (SELEX G) on the activity of their cognate enzyme.** Activity of SMD-Ll1 or SMD-Ll2 in the presence of all pools of RNA obtained from SELEX E (top, Ll1), SELEX F (middle, Ll1) or SELEX G (bottom, Ll2) at SMD:RNA molar ratios of 1:1 was calculated from fluorescence measurements. SMD activity is expressed as percent of the fluorescence counts obtained with each protein in the absence of RNA (black bars). Data shown are averages of three experiments (n = 3), each in triplicate (SELEX E and F) or duplicate (SELEX G), ± standard error of the mean (SEM). Significant differences (P < 0.001), determined by ANOVA of repeated measures and the Tukey post-test, are shown as §, relative to protein alone in the absence of RNA; *, relative to s1; and †, relative to s12.

The sphingomyelinase activity of recombinant toxins was measured in the presence of the RNA recovered from each selection round of SELEX E, F, and G (s1 through s12) to determine its apparent inhibitory capacity; assays were done using protein:RNA molar ratios of 1:1. In all cases (E, F and G) (Figure 2) sphingomyelinase activity in the presence of RNA from the initial cycles (s1-s3) was greater than that of protein alone. SELEX E (Ll1) and SELEX G (Ll2) behaved similarly in that the activity obtained with s1 RNA dropped gradually along the first 4–8 cycles followed by an increase in activity in cycle 9 and thereon (more pronounced and significant in E). For SMD-Ll1 (SELEX E), activity reaches a 9% maximum reduction on s8 (25% reduction between s1 and s8, P < 0.001). For SMD-Ll2, activity also reaches a maximum reduction (3.3%) on s8 (17.6% reduction between s1 and s8, P < 0.001). Accordingly, RNA pools Es8 and Gs8 were chosen for cloning to isolate and characterize individual aptamers with therapeutic potential. RNA pools Es12 and Gs12 were also cloned; their binding affinity is expected to be greater than that of s8 RNAs and, therefore, they may have greater potential for diagnostics. SELEX F (Ll1), in which the selection pressure was less than that applied in SELEX E, allowed reaching a 25% reduction (P < 0.001) of SMD-Ll1 activity with s10 RNA (last round performed), suggesting that even more profound effects might be attained in additional selection rounds.

### Sequence characterization of individual aptamers

The DNA pools encoding RNA molecules from selection rounds Es8, Es12 and Fs10 (selected for binding to SMD-Ll1), and selection rounds Gs8 and Gs12 (selected for binding to SMD-Ll2), were cloned in pUC19. Individual clones (~50 from each pool) were sequenced, and reliable sequences (on average 90%) were analyzed to establish families of related aptamer sequences. Taking into account the 60 random nucleotides in the center of each 107 nt aptamer, sequence identity within a family was > 92% (*i.e.*, less than 5 nt differed between family members, typically 2 or 3), while identity between families was < 60%. Sequences entirely different from all others in that cohort were termed orphan sequences.

Analysis of cohort Es8 revealed 49/54 clones (91%) grouped in 6 families, and 5 orphan sequences; of 26 unique sequences the most frequent one was found in 9 clones. Similarly, cohort Es12 had 36/40 clones (90%) belonging to 7 sequence families, and 4 orphan sequences. Of 24 unique sequences the most frequent one was found in 5 clones, and 7 (29%) were also present in cohort Es8. Cohort Fs10 had 42/47 clones (89%) that could be grouped in 9 sequence families, and 5 orphan sequences. Of 32 unique sequences the most frequent one was found in 6 clones, and 11 (34%) had already been found in SELEX E: Fs10 shares 7 sequences with both Es8 and Es12, 2 with Es8 only, and 2 with Es12 only. Thus, independent selection processes E and F, both performed with SMD-Ll1 and starting from the same ~10^13^ RNA variants, led to common aptamers.

As for the iterative selection performed by affinity for SMD-Ll2, analysis of cohort Gs8 revealed 35/49 clones (71%) grouped in 11 sequence families, and 14 orphan sequences; of 37 unique sequences the most frequent one was found in 8 clones. Similarly, cohort Gs12 had 42/52 clones (82%) belonging to 9 sequence families, and 10 orphan sequences. Of 29 unique sequences the most frequent one was found in 13 clones, and 8 (28%) were also present in cohort Gs8.

As expected, no sequences were found to be common between the SMD-Ll1 and the SMD-Ll2 selections; SELEX E (and F) and SELEX G were initiated with RNA pools that were theoretically entirely different given that they originated from different aliquots of the random oligonucleotide synthesis. Es8, Fs10 and Gs12 were chosen for functional characterization of individual aptamers.

### Functional characterization of individual aptamers

**Figure 3:**
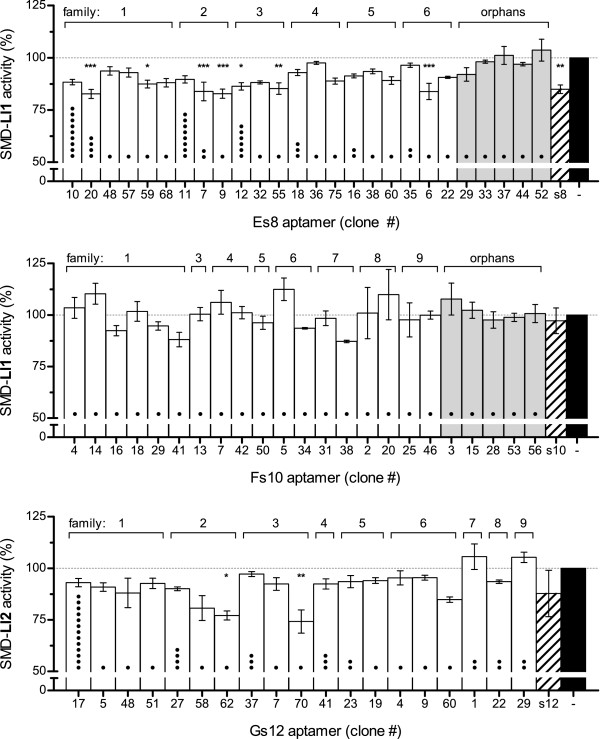
**Effect of individual RNA aptamers from the Es8, Fs10, and Gs12 cohorts on cognate SMD activity.** Activity of SMD-Ll1 and SMD-Ll2 in the presence of RNA aptamers found in cohorts Es8 (top, Ll1), Fs10 (middle, Ll1), and Gs12 (bottom, Ll2) at SMD:RNA molar ratios of 1:10 was calculated from fluorescence measurements. SMD activity is expressed as percent of the fluorescence counts obtained with each protein in the absence of RNA (black bars). Data shown are averages of three experiments (n = 3), each in triplicate, ± SEM. The inhibitory effects of the RNA pools of origin are shown in hatched bars. The dots (•) in each bar represent the number of clones that were found to have the same sequence as that specific clone. Clones belonging to families are shown in white bars and orphan clones are shown in grey. Significant inhibitions relative to protein alone were determined by an ANOVA of repeated measures and the Tukey post-test (*** P < 0.001, ** P < 0.01, * P < 0.05).

All unique aptamers (either orphans or belonging to sequence families) found in cohorts Es8 and Gs12 were tested for their capacity to inhibit the enzymatic activity of their cognate sphingomyelinase; Fs10 aptamers not found in Es8 were also assayed. The SMD:RNA molar ratio used in this inhibition assay (Figure 3) was 1:10, both for individual aptamers as well as for their pools of origin.

In cohort Es8 (Figure 3, top panel) there were four aptamers that elicited 16–17% inhibition of SMD-Ll1 (P < 0.001); none of the orphan aptamers elicited a significant reduction in sphingomyelinase activity. The sequences of these leading aptamers, Es8-6 (family #6), Es8-7 and Es8-9 (family #2), and Es8-20 (family #1) were not the most abundant in the group, but three of them belong to the two most populated families in Es8. Three of these aptamers arose independently in both SELEX E and SELEX F, namely, Es8-6, Es8-7, and Es8-20, one from each family. These aptamers elicit inhibitions that are very similar to or greater than the apparent inhibitory capacity of the Es8 RNA pool or the Fs10 pool (~17% *vs.* 15% or 3% respectively). None of the aptamers unique to Fs10 displayed significant reductions of SMD-Ll1 activity (Figure 3, middle panel).

In cohort Gs12 (Figure 3, bottom panel) there were two aptamers that elicited ~23% and ~26% inhibition of SMD-Ll2 (P < 0.05 and P < 0.01); none of the orphan aptamers elicited significant reductions in sphingomyelinase activity (data not shown). Although the two leading aptamers —Gs12-62 (family #2) and Gs12-70 (family #3)— were found in only one copy amongst the 52 clones sequenced, they belong to the second and third most populated families in Gs12, two families which had already arisen in Gs8. As expected for some individual aptamers, these leaders elicit inhibitions greater than that of the Gs12 RNA pool (~25% *vs.* 12%).

Worth noting is that RNA pools elicit different inhibitions of sphingomyelinase activity depending on the SMD:RNA ratio used in the assay, 1:1 (Figure 2) or 1:10 (Figure 3). This may be explained by the fact that these are pools of many RNAs, each having a unique combination of binding affinity and inhibitory capacity and each represented in a different number of copies; the possible RNA-RNA interactions in each pool are likewise unique. Depending on which set of aptamers coexist in a given pool, the overall behavior is as likely to change in one direction or the other when the SMD:RNA ratio is changed.

**Figure 4:**
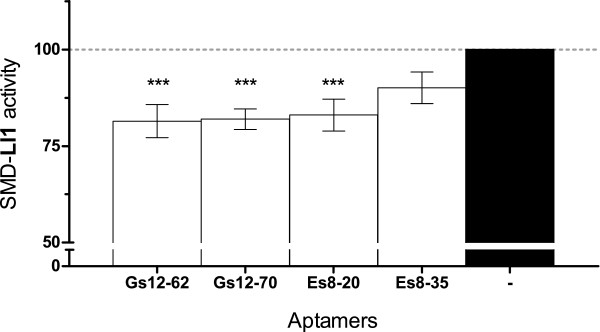
**Cross effects of leader Gs12 aptamers on SMD-Ll1 activity.** Activity of SMD-Ll1 in the presence of RNA aptamers selected for binding to SMD-Ll2 (Gs12-62 and Gs12-70) at SMD:RNA molar ratios of 1:10 was calculated from fluorescence measurements. SMD activity is expressed as percent of the fluorescence counts obtained with SMD-Ll1 in the absence of RNA (black bar). The effects of control aptamers selected for binding to SMD-Ll1 are also shown: Es8-20 (inhibitory RNA), and Es8-35 (noninhibitory RNA). Data shown are averages of six experiments (n = 6) except for Es8-35 (n = 3), each in triplicate, ± SEM. Significant inhibitions relative to protein alone were determined for data in n = 6 by an ANOVA of repeated measures and the Tukey post-test (*** P < 0.001).

An interesting question to address is whether the best two inhibitory aptamers, those selected for binding to SMD-Ll2, are capable of inhibiting a noncognate SMD. Cross activity experiments (Figure 4) show that aptamers Gs12-62 and Gs12-70 clearly exert a significant inhibitory effect on SMD-Ll1 (~18%).

## Discussion

The original gene constructs from which SMD-Ll1 and SMD-Ll2 were produced in *E. coli*[13], were not available for this work. Hence, *de novo* synthesis was adopted to generate the cDNAs. Once cloned in the expression plasmid pQE30-Xa, akin to what was reported in the literature, no bacterial production of the enzymes was observed. However, recloning in a different vector yielded the recombinant proteins. Specific activities (± SD) were approximately 17 ± 6 U/mg for SMD-Ll1 and 159 ± 9 U/mg for SMD-Ll2, somewhat lower than those reported by Olvera *et al.*[13] for recombinant SMD-Ll1 (also tagged in the N terminus), and for SMD-Ll2 tagged in the C terminus, 56.8 ± 9.9 and 228.2 ± 58.7 U/mg respectively. Thus, this is the first report of an active recombinant SMD-Ll2 bearing the histidine tag on the N terminus; this variant was expected to be active because the crystallographic data available [25] show both protein ends unencumbered. Initial attempts to remove the histidine tags with factor Xa to preclude any interference during SELEX were not quantitative but optimization efforts were not pursued because preliminary studies showed that aptamer pools with inhibitory activity were obtained, suggesting that the tags did not hinder selection of RNA aptamers based on their recognition by the SMD portion of the recombinant proteins.

The sphingomyelinase activity inhibition data for selected RNA pools show that there are statistically significant changes in RNA behavior as selection rounds proceed, suggesting that selection took place along the process, even though apparent affinity of selected RNA pools was not measured. When selection pressure was defined by a 1:10 protein:RNA molar ratio, a net inhibition of SMD activity became apparent with s4 RNA, reached a maximum with s8 RNA, and was lost or remained stagnant (E and G respectively) as selection proceeded, that is, as apparent affinity is expected to increase. A reasonable explanation for this behavior, as well as for the activating effect seen with RNA from the initial rounds (s1-s3), is that both activating and inhibiting aptamers exist in the selected pools; given that the selection was carried out by affinity, some activating or noneffector aptamers may bind more tightly than inhibitory aptamers, eventually displacing the latter. Hence, maximum affinity pools are probably not enriched in the best sphingomyelinase inhibitors. It follows that if the selection processes had been monitored by affinity the best inhibitor pools might have been overlooked. Given that a good inhibitor necessarily has to bind, this work suggests that a feasible route to obtain an aptamer with good therapeutic potential is selection by affinity, followed by identification of inhibitors, and then by optimization of inhibition and/or binding capacities.

The maximum inhibitory capacity obtained was ~26% (aptamer Gs12-70), far from the full inhibition that is naturally desirable in an antidote. Bearing in mind that selections were done on the basis of binding —not inhibitory capacity— several additional reasons may explain this outcome. First, as seen from comparing selection processes E and F (Figure 2), a less competitive and slower SELEX may be better; a protein (SMD-Ll1):RNA ratio of 1:5 allowed the Fs10 pool to exert an inhibition of 25%, greater than the maximum inhibition obtained when the selection ratio was 1:10 (9% for Es8). Thus, many good candidates may have been lost in the early stages of the E process. Second, sequencing a greater number of clones per cohort, *e.g.,* 100 clones, might have allowed the identification of stronger inhibitors. Third, although the initial DNA pool (s0) was subjected to digestion with *Xba*I to avoid selecting clones that would be lost in the final cloning step, this was not done with *Hin*dIII, the other restriction enzyme used for cloning. Thus, good inhibitors might have been lost upon cloning. These observations offer guidelines for future efforts to obtain aptamer antidotes for these SMDs. An alternative route to obtaining aptamers with greater inhibitory capacity is to optimize the effects of the best available (partially inhibitory) RNAs; the finding that two aptamers that differ solely in one nucleotide, *e.g.*, Gs12-37 and Gs12-70, have different inhibitory capacities, suggests that mutagenesis of leader aptamers might be worthwhile. A SELEX approach starting from a leader RNA subjected to mutagenesis in its entire sequence or in only one or several nucleotides, may be considered.

## Conclusions

The application of SELEX technology to obtain therapeutic or diagnostic agents for biological toxins has been reviewed recently [26]. It is a very promising approach given the advantages inherent to the small size and chemical nature of nucleic acid aptamers when compared to antibodies. Additionally, nucleic acid aptamers may be coupled to various detection methods and are thus very versatile as diagnostic agents [27]. In this work, six RNA aptamers capable of partial inhibition (16-26%) of two *Loxosceles laeta* sphingomyelinases, SMD-Ll1 and SMD-Ll2, were obtained by iterative selection, constituting an initial platform for the development of novel nucleic acid drugs that may be both safe and specific for the venom of the most dangerous species of the *Loxosceles* spiders. To our knowledge, this is the first attempt to obtain aptamers with therapeutic and diagnostic potential for loxoscelism.

## Methods

### Synthesis of cDNAs and gene constructs encoding *Loxosceles laeta* SMD isoforms

Gene expression constructs encoding recombinant *L. laeta* SMDs Ll1 and Ll2 were generated. The 858 bp cDNAs for the mature form of SMD-Ll1 [GenBank:DQ369999] and SMD-Ll2 [GenBank:DQ37000] [13] were assembled *in vitro* by polymerase chain assembly, which employs long synthetic oligonucleotides as starting material for extension and amplification reactions [28]; Pfu DNA polymerase (Fermentas) was used. For each SMD, 22 overlapping oligonucleotides were designed (overlaps of 18 nts): 2 external oligonucleotides (33 nts each) and 20 internal oligonucleotides (59 nts each), all synthesized by Integrated DNA Technologies (IDT). Cloning schemes demanded introducing single nucleotide modifications as well as the addition of AAG CTT ACT G to the 3′ end to incorporate a *Hin*dIII site. Specifically, for SMD-Ll1, a nonsilent mutation 26A > T (N9I), and four silent mutations (36 T > C, 462 T > C, 618C > T, 837A > T); for SMD-Ll2, five silent mutations (141 T > C, 300 T > C, 462 T > C, 618C > T, 837A > T). Each cDNA was assembled in two halves from which full-length sequences (868 bp) were obtained. Amplicons were purified from agarose gels with the Wizard SV Gel and PCR Clean-up System (Promega). The sequences required for protein production and purification were provided by directional cloning (blunt end/*Hin*dIII) in pQE30-Xa (Qiagen) (*Stu*I, Klenow, *Hin*dIII). The *Eco*RI-*Hin*dIII fragments from these intermediate constructs contained sequences encoding a ribosome binding site, an initiation codon, and a recombinant protein having a His_6_ tag, the four residues required for proteolytic cleavage by factor Xa, and a mature SMD. These fragments were transferred to pGEM-3Zf(+) (Promega) downstream of the T7 RNA polymerase promoter (*Eco*RI/*Hin*dIII) to generate final gene expression constructs pCC-8 and pCSL-2 encoding SMD-Ll1 and SMD-Ll2 respectively. Plasmids were purified from *E. coli* DH5α cultures (Wizard Plus SV Minipreps DNA Purification System, Promega) and constructs were confirmed by sequencing (CESAT, Universidad de Chile).

### Production and purification of recombinant SMD-Ll1 and SMD-Ll2

Recombinant SMD-Ll1 and SMD-Ll2 were produced in *E. coli* Origami B (DE3) (Novagen) transformed with pCC-8 or pCSL-2 respectively. Luria broth cultures (50 mL, 100 μg/mL ampicillin) were grown in 250 mL flasks at 37°C and 250 rpm to an A_600_ of 0.5-0.7 (4.5-5 h). Gene expression was induced with 1 mM IPTG and, after 16 h of growth, 35 mL of culture were harvested by centrifugation at 4°C. Cells were resuspended in 5 mL of NPI-10 (50 mM NaH_2_PO_4_, 300 mM NaCl, 10 mM imidazole) and lysed by ≥ 4 freeze-thaw cycles (−80°C freezer/37°C water bath, 30 min each). DNaseI was added (10 μg/mL) and lysates were incubated 30 min in a 37°C water bath, clarified by centrifugation (12857 × g, 30 min, 4°C) and filtered through polyethersulfone membranes (Millex GP, 0.22 μm, Millipore).

**Figure 5:**
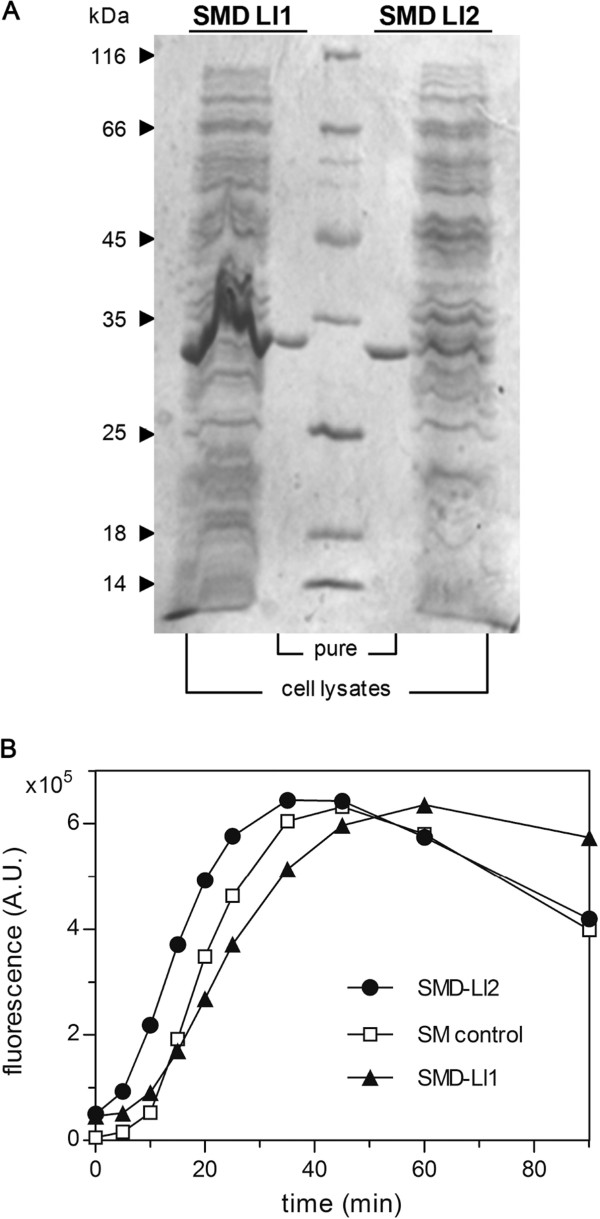
**Recombinant SMD preparations: purity and activity.** The sphingomyelinase activity of pure His_6_ tagged recombinant toxins SMD-Ll1 and SMD-Ll2 purified from *E. coli* lysates was measured with a fluorescent method employing the Amplex Red reagent in a microplate reader instrument. **A**. SDS-polyacrylamide (10%) gel stained with Coomassie blue showing bacterial lysates and purified fractions of both SMD-Ll1 and SMD-Ll2. **B**. Activity curves: the fluorescence of resorufin is shown in arbitrary units (A.U.) through time. Data shown are for 100 ng SMD-Ll1; 20 ng SMD-Ll2, and 4 mU of control sphingomyelinase (*Bacillus cereus*).

Recombinant proteins were purified by affinity chromatography using 1 mL size Ni-NTA Superflow cartridges (Qiagen). After loading, columns were washed with 10 volumes of NPI-20 (20 mM imidazole) and bound proteins were eluted with 10 mL of NPI-250 (250 mM imidazole) in 0.5-1 mL fractions. Purifications were monitored in 0.1% SDS-10% polyacrylamide gels and pure fractions were dialysed in 3 mL Slide-A-Lyzer cassettes (10,000 MWCO) (Pierce) against 50 mM Tris–HCl pH 8 (Figure 5A). The Micro BCA Protein Assay Kit (Pierce) was used for quantification in flat bottom clear 96 well plates (Costar 3635) using a bovine serum albumin (BSA) standard curve; absorbance was measured after 2 h at 37°C in a Victor X3 (Perkin Elmer) plate reader (560 ± 8 nm). Approximate yields were 6 mg for SMD-Ll1 and 0.5 mg for SMD-Ll2. Both 306 aa recombinant proteins, of 34.2 and 34.8 kDa respectively, have a synthetic 21 aa peptide (MRGSH_6_GSGSGSGIEGR) preceding the 285 residues of mature toxin sequence.

### Sphingomyelinase activity measurements

Activity of recombinant proteins was measured in 100 mM Tris–HCl pH 7.4, 10 mM MgCl_2_ with the Amplex Red Sphingomyelinase Assay Kit (Invitrogen) in 200 μL reactions (100 μL of sample plus 100 μL working solution). Fluorescence of resorufin was read in white flat bottom 96 well plates (Nunc 236108) in a Victor X3 instrument for 60–90 minutes (excitation: 544 ± 15 nm, emission: 590 ± 20 nm). A linear behavior between activity and fluorescence was generally obtained throughout the first 20 minutes (Figure 5B) with 100 and 20 ng of SMD-Ll1 and SMD-Ll2 respectively; activities were calculated at 10, 15 or 20 minutes.

### Generation of the initial random RNA pool

**Figure 6:**
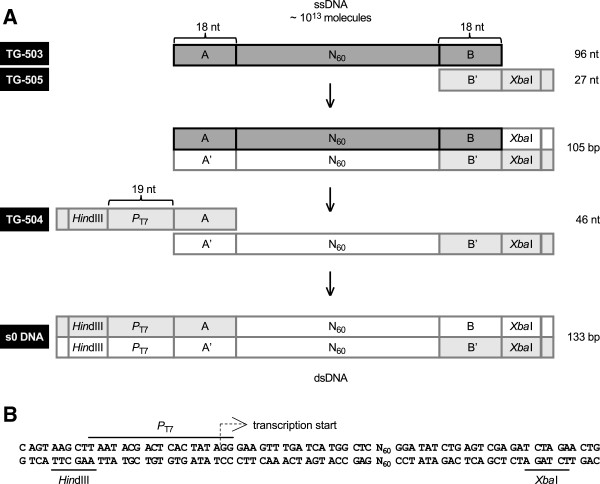
**Synthesis of random sequence template DNA (s0) by PCR. A**. Amplification of oligonucleotide TG-503 (96 nt) by PCR with primers TG-504 (46 nt) and TG-505 (27 nt); the template has a 60 nt central region of random sequence. The resulting s0 DNA (133 bp) has a promoter for T7 RNA polymerase, which allows the generation of RNA by *in vitro* transcription, and recognition sites for restriction enzymes *Hin*dIII and *Xba*I, which allow cloning for the analysis of individual sequences. **B**. Nucleotide sequence of s0 DNA.

Suitable templates for *in vitro* transcription were generated as follows (Figure 6). A long DNA oligonucleotide (TG-503, 96-mer) having a 60 nt center of random sequence flanked by segments A and B was synthesized (GAA GTT TGA TCA TGG CTC N_(60)_ GGA TAT CTG AGT CGA GAT). A full-length degenerate duplex (133 bp) was obtained by PCR amplification of 10 pmoles of TG-503 (5 U Biolase Taq DNA polymerase (Bioline), 200 μM each dNTP, 10% dimethylsulfoxide, 1.5 mM MgCl_2_; 2 min 94°C/30 cycles of 30 sec 94°C, 30 sec 65°C, 2 min 72°C/10 min 72°C) using primers TG-504 and TG-505 (100 pmoles each) in ten 50 μL reactions. Oligonucleotides TG-503, 504 and 505 (IDT) were designed according to Sapag and Draper [29] with modifications. Primer TG-504 (CAG TAA GCT TAA TAC GAC TCA CTA TAG GGA AGT TTG ATC ATG GCT C, 46-mer) anneals to flanking sequence A, provides the T7 phage RNA polymerase promoter and a *Hin*dIII recognition sequence, while primer TG-505 (CAG TTC TAG ATC TCG ACT CAG ATA TCC, 27-mer) anneals to flanking sequence B and carries an *Xba*I recognition sequence. The degenerate amplicon was digested with *Xba*I and purified from a 3% Nusieve agarose gel.

Transcription was done at 37°C in 50 μL reactions for 2 hours using ~250 ng of degenerate duplex, 20 U T7 RNA polymerase (Fermentas), and 2 mM each NTP. Products were precipitated and separated by electrophoresis in 8% denaturing (50% urea w/v) polyacrylamide gels run at 17 W for 1 h. Full-length RNAs were visualized by UV-shadowing, bands were excised and soaked in water overnight at 37°C, and RNAs (102 nt) were concentrated with Amicon Ultra (2 mL 30 K) centrifugal filters. RNA concentrations were measured by absorbance at 260 nm.

### Selection cycles

Protein and RNA were prepared separately for binding in equal volumes (25 μL) prior to mixing (50 μL). Typically, 10 pmoles of toxin (~0.3 μg) and 100 pmoles of RNA (~3.3 μg) were used (1:10 molar ratio). The protein was incubated in 100 mM Tris–HCl pH 7.4 for 20 min at 37°C and 10 min at room temperature. The RNA was renatured in 100 mM Tris–HCl pH 7.4 and 20 mM MgCl_2_ for 20 min at 42°C and 10 min at room temperature. After combining the two, binding conditions being 100 mM Tris–HCl pH 7.4 and 10 mM MgCl_2_, mixtures were incubated for 10 minutes at room temperature. Filtration through nitrocellulose membranes (Whatman # 7182–002, 2.5 cm) allowed the separation of RNA molecules that were bound to the protein (retained on the filter) from those that were unable to bind. Filters were washed twice with 200 μL of binding buffer. The bound (selected) RNAs were recovered from the filter (cut into 2×2 mm pieces) by soaking for 1 h at room temperature in 200 μL of 10 mM Tris–HCl pH 7.4 and 400 μL of water saturated phenol; RNAs were precipitated using 20 μg of glycogen (Roche) as carrier and resuspended in water.

RNAs selected in each cycle were reverse transcribed in one 20 μL reaction. Briefly, the RNA was incubated in standard buffer with 0.5 mM each dNTP and 100 pmoles TG-505 for 10 min at 55°C and switched to ice prior to the addition of 200 U of M-MLV reverse transcriptase (Promega) and incubation at 42°C for 1 h. Following precipitation, the entire cDNA preparation was amplified in 5 PCR reactions with primers TG-504 and TG-505. The DNA was pooled, precipitated, and full-length molecules were purified from a 3% Nusieve agarose gel. Enough RNA for the next selection step was generated from this DNA (which was not subjected to *Xba*I digestion) in 5 *in vitro* transcription reactions, each with 250 ng of template. Reactions were pooled, nucleic acids were precipitated, and the full-length RNA (107 nt) was purified from polyacrylamide gels, concentrated, and used for the next selection cycle.

### Inhibition of sphingomyelinase activity by pools of selected RNA

Binding reactions were performed as indicated for the selection steps but at protein:RNA molar ratios of 1:1, using 100 ng of SMD-Ll1 and 20 ng of SMD-Ll2 per enzymatic assay. Enough protein for a group of assays was prepared for binding in one tube (25 μL per assay) while RNAs from different selected pools were renatured individually in one tube per assay (25 μL). The protein was added to the RNA and, after the 10 min binding step, the protein-RNA mixture was added to 50 μL of binding buffer in each well of a 96-well plate, making 100 μL of sample over which 100 μL of Amplex Red working solution were added to initiate the enzymatic reaction. Sphingomyelinase activity was measured as indicated. Typically, 3 experiments (n = 3) were performed in triplicate. For each experiment, the average of blank fluorescent counts was subtracted from individual measurements; triplicates were averaged and expressed as percent of the activity obtained for the protein in the absence of RNA (100% activity) in that same experiment. Data were subjected to analyses of variance (ANOVA) of repeated measures and the Tukey post-test (GraphPad Prism software).

### Cloning of RNA pools and sequencing of individual clones

The cDNAs of some RNA pools were cloned in pUC19 (New England Biolabs) using *Hin*dIII and *Xba*I (full transcription units). Clones were sequenced with M13 universal primers in microplate format (Macrogen, USA). Sequence relatedness between clones was analyzed with Clustal W 2.1 [30]. Families of sequences (groups comprising two or more clones) were named with increasing numbers (starting with family #1 in each cohort) according to the following hierarchy: total number of clones, number of identical clones, lowest clone number (clone name).

### Inhibition of sphingomyelinase activity by individual RNA aptamers

Performed as detailed for pools of selected RNA but at protein:RNA molar ratios of 1:10. Each RNA was generated in several 50 μL *in vitro* transcription reactions using 250 ng of template. Duplex templates were obtained by PCR performed on each plasmid clone with primers TG-504 or M13-reverse and TG-505 using 1 mM MgCl_2_ (2 min 94°C/35 cycles of 1 min 94°C, 2 min 60°C, 2 min 72°C/10 min 72°C). Amplicons were precipitated prior to use as templates. Typical yields were 1 μg of DNA per reaction and 2–8 μg RNA per transcription. Full length RNAs were purified as indicated. Large amounts of RNA were alternatively obtained with the TranscriptAid T7 High Yield Transcription Kit (Thermo Scientific). Typically, a single 20 μL reaction was carried out for 18 hours at 37°C using 250 ng of PCR product as template; yields were 20–80 μg of RNA. Following purification, RNAs were concentrated by precipitation.

## Abbreviations

ANOVA: Analysis of variance
PCR: Polymerase chain reaction
SELEX: Selection of ligands by exponential enrichment
SEM: Standard error of the mean
SMD: Sphingomyelinase D.
